# Microwave-assisted hydrogen peroxide digestion followed by ICP-OES for determination of metals in selected fuel oils

**DOI:** 10.1038/s41598-024-52898-4

**Published:** 2024-01-29

**Authors:** Njabulo S. Mdluli, Cyril D. Knottenbelt, Philiswa N. Nomngongo, Nomvano Mketo

**Affiliations:** 1 Department of Chemistry, College of Science and Engineering and Technology, Florida Science Campus, University of South Africa, Roodepoort, Johannesburg, 1710 South Africa; 2 PetroSA, Private Bag X14, Mossel Bay, 6520 South Africa; 3 Department of Chemical Sciences, University of Johannesburg, PO Box 17011, Doornfontein, Johannesburg, 2028 South Africa

**Keywords:** Environmental sciences, Chemistry

## Abstract

This work describes a greener and cost-effective microwave-assisted hydrogen peroxide digestion (MA-HPD) with the addition of 1 mL of HNO_3_ (70% v/v) to enhance extraction of selected metals (Al, Ba, Cd, Co, Cr, Cu, Mg, Na, Ni, Pb, Sb, Ti and V) in crude-oil, diesel, gasoline and kerosene samples prior to inductively coupled plasma-optical emission spectroscopic (ICP-OES) analysis. The most influential parameters of the MA-HPD method were investigated by using multivariate optimization tools (two-level full factorial and central composite designs) and fuel oil certified reference material (NIST1634c). The optimum conditions were observed to be 245 ℃ microwave temperature, 25 min digestion time, 0.1 g sample mass and 5 M H_2_O_2_ were the optimum digestion conditions with accepted accuracy (104.8–117.7%) and precision (≤ 4.1%). In overall, the metals that reported high concentrations in the crude oil, diesel, gasoline, and kerosene samples were Na (51.94–58.86 mg/kg) and Mg (36.08–47.4 mg/kg), while Cu was the lowest (0.55–2.89 mg/kg). When comparing the obtained concentration levels with other literature reports, a conclusion can be drawn that South Africa is importing oils of reasonable quality.

## Introduction

As a refinery raw material crude oil continues to dominate the South African refinery scene, South Africa imports between 17,000 and 25,000 tons of crude oil per annum for refining to final product automotive fuels^1^. Crudes can be sourced from a number of sources around the world and the qualities of the crude will vary greatly from regions. The hydrocarbon class types that are found in crudes are paraffinic, naphthenic, aromatic and asphaltic^2^. Crudes with high metal content are undesirable since the metals can act as catalyst poison, reducing catalyst activity which result in slowed refinery process^3^. Metals such as nickel, iron and vanadium have a long history of being problematic to refiners^4^. Alkali metals can form metal soaps and lead to unwanted emulsions and inability to separate hydrocarbon from aqueous phases^5^. Chloride containing salts present in the crude oil could be hydrolysed to HCl and result in severe refinery corrosion^6^. The quality of the crude oil has a profound impact on refining and final quality of products (crude oil fractions such as diesel, gasoline and kerosene).

Crude oil is a natural energy resource that is mainly composed of hydrogen and carbon based organic compounds and minor inorganic components^2^. The latter include multi-elements such as metals and non-metals^5^. Some of these metals are detrimental to living organisms, environment and can result in reduced lifespan of refinery equipment and cause harm to internal combustion motor vehicle engines^5^. Corrosion makes the refinery machine less effective, thus reducing its lifespan^2^. The problem associated with corrosion of refinery equipment is contamination as corroded materials mix with the crude oil. The maintenance and repairing of the refinery machines may be very costly and the huge expense on oil refinery maintenance in turn negatively influence the market price of the petroleum products^2^. Additionally, Cd, Hg, Pb and As are associated with air pollution, thereby affect living organisms^6^. Alternatively, Ni, V, Pb, Pt and As are known to be catalyst poisoners during refinery process. Crude oil is then refined to form crude oil fractions like gasoline, diesel, kerosene, just to name the few. When gasoline and diesel are combusted, the presence of Cu, Fe, Co and Mn can catalyse the oxidation reaction, thereby causing low combustion efficiency, resulting in unburnt deposits and also cause metal laydown in the cylinder^3^. Therefore, the challenges associated with metals in crude oil and crude oil fractions have ignited an interest for many researchers to conduct investigations on the development of analytical methods for quantitative determination of metals in various fuel oils.

Literature reports have proved that inductively coupled plasma based techniques (ICP-OES and ICP-MS) are the best option for elemental analysis, due to their multi-elemental capabilities and low detection limits^7^. However, fuel oils contain high carbon content and therefore they require mineralization method prior to spectrometric analysis of metal ions^8^. Therefore, several digestion methods have been reported for mineralization of oily matrices. These methods include, microwave assisted acid digestion (MAAD)^7^, microwave assisted-single reaction chamber (MA-SRC)^9^ and microwave induced combustion (MIC)^10^. However, literature reported several limitations associated with the above-mentioned sample preparation methods. For example, the use of concentrated HClO_4_ and H_2_O_2_ is associated with explosive conditions^11^, concentrated HNO_3_ produce carcinogenic nitrous oxide^12^, while HCl and HF are corrosive and can dissolve glass optics of the spectrometric techniques and are highly toxic^13^. Acid waste generated can cause environmental pollution^14^ and concentrated acids can be costly during MAAD^15^. Additionally, resulted digest containing concentrated acids can cause matrix effect challenges during spectrometric quantification using external aqueous calibration standardization method^16^. On the other hand, MIC makes use of dilute acids, therefore overcome limitations of MAAD. The main limitations of MIC are the use of expensive oxygen, quartz vessels and the additional reflux step, which prolongs the digestion^8^. Lastly, MASRC make use of one digestion vessel at a time, which is a huge disadvantage when running a large population sample^17^. In 2015, the use of a novel and green microwave assisted-hydrogen peroxide digestion (MA-HPD) method followed by ICP-OES and ion- chromatographic for quantitative determination of total sulphur in coal samples was developed^18^. This sample preparation method corrected some limitations of MAAD, which included, production of hazardous waste, matrix effects caused by concentrated acids and was proven to be cost-effective and environmentally friendly. This is because at temperatures above 150.2 ℃, diluted H_2_O_2_ used gets converted to H_2_O and O_2_ during digestion (see Eq. 1). The ICP-OES results showed accepted sulphur recoveries of 89–102%, excellent precision of ≤ 1.5% and low detection limits of 0.014 µg/g. Another study on the use of dilute H_2_O_2_ and dilute HNO_3_ was reported for the digestion of coal samples for quantitative extraction of trace elements with low detection limits of detection (0.003–3.5 µg/g) and high accuracy (92–114%) for most of the investigated elements^8^.

1 $$2H_2O_{2(aq)} \stackrel{\Delta 150.2^\circ{\rm C} }{\to } 2H_2O_{(1)} +O_{2(g)}$$

For the optimisation of the best digestion parameters, multivariate optimisation have been used over univariate optimization. During univariate optimisation a single parameter is optimised at a time while keeping the other parameters constant^19^. In contrast, multivariate optimization investigates several parameters simultaneously and this helps to save time and reduce reagents used as less experiments are conducted in comparison to univariate^20,21^. The two-level full factorial was used for screening and the central composite design was used for response surface methodologies (RSM). The central composite design was chosen based on its easy operation when compared to the other RSM^22^. Additionally, multivariate in microwave assisted digestion was also reported by Barela et al*.*^20^, where a two-level full factorial was only used to optimize for [HNO_3_] and [H_2_O_2_] prior to analysis Ba, Co, Cr, Cu, Mn, Ni, Pb, Sr and V in biodiesel samples using Sector Field Inductively Coupled Plasma Spectrometry (SF-ICP-MS). The expression for a full factorial is 2^n^, where n is the number of parameters to be optimized.

Therefore, the current study proposed the use of multivariate optimization procedure for optimization of microwave digestion parameters (time, temperature, H_2_O_2_ concentration and sample mass). It is worth noting that for the first time a much dilute HNO_3_ (1.57 M) reagent was used, which resulted in an approximately 0.63 M acid for the 25 mL final digest. This makes the proposed mineralisation method to be green, since the generation of secondary waste normally caused by concentrated acids will be eliminated. Additionally, crude oil samples that are imported to one of the South African crude oil refinery stations were investigated. Lastly, the study also aimed at evaluating if South Africa is importing quality oils.

## Experimental methods

### Reagents and glassware

All reagents used were of analytical grade purity and Milli-Q water obtained from a water purification system (USA**)** with water resistivity of 18.2 MΩ cm, which was used for rinsing and making up solutions. Multi-element standard of 100 mg/L (purchased from Sigma-Aldrich, South Africa) was used in the preparation of different concentrations of standard solution**.** Standard reference material (SRM/NIST1634c) for trace elements in fuel oil was also purchased from Sigma-Aldrich, South Africa. The 70% (v/v) ACS grade HNO_3_, and Suprapure 30% (v/v) H_2_O_2_ were purchased from Merck, South Africa.

Crude oil samples were obtained from a petrochemical company. Hydrocarbon final products comprising of diesel, gasoline, and kerosene were purchased in three different filling stations labelled as A, B and C respectively around Johannesburg in South Africa. Polyvinylidene difluoride (PVDF) microfilters size of 0.45 µm pore diameter were purchased from Anatech instrument (South Africa). All glassware were washed using soapy water, then soaked in 5% nitric acid solution for 24 h, rinsed with deionized water and allowed to dry in the oven (EcoTherm Labotec) for overnight.

### Instrumentation

An Anton Paar Multiwave 5000 microwave digester was used for digestion of fuel oil samples. The microwave was equipped with a rotor (20SVT) which holds a total of 20 polytetrafluoroethylene-Teflon vessels (PTFE-TFM) at a time. It is worthy to indicate that any of the vessels can be used as a reference unlike in some microwaves where only the vessel with a temperature probe is used as a reference. The microwave was equipped with a temperature programme where the ramping and holding durations were controlled. The microwave system was set to ramp for 10 min to 245 °C and holding time was 15 min at 245 °C. After the holding time of 15 min, the microwave was allowed to cool to 70 °C and the vessels were removed from the rooter for further cooling until they were at room temperature. The resulted digests were analysed for metals by using Agilent Technologies 700 Series ICP-OES with an axial orientation of the torch. Additionally, an Agilent Technologies SPS 3 autosampler was used for sample uptake while a concentric nebulizer and a cyclonic spray chamber was used for sample introduction. The optimum operational conditions for analysis are presented in Table 1.

**Table 1:** Operating parameters of the Agilent Technologies 700 Series ICP-OES for metal and metalloids analysis.

Agilent ICP-OES instrumental parameters	Condition
RF Power	1200 W
Auxiliary gas Flow	1.5 L/min
Plasma gas (Ar) flow rate	15.0 L/min
Pump speed	85 rpm
Peri-pump speed analysis	15 rpm
Sample uptake delay (s)	15 s
Stabilization time (s)	15 s
Nebulizer	0.75L/min
Elemental wavelengths (nm)	Al 396.52, Ba 234.759, Co 201.151, Cr 206.550, Cu 327.395, Ni 216.55, Mg 279.553, Na 588.995, Pb 283.30, Sb 217.582, Ti 336.122, and V 292.299

### Microwave assisted hydrogen peroxide digestion (MA-HPD)

Microwave digestion system uses microwave energy to break the carbon and metal bond^23^. This is a form of energy that is non-ionizing from electromagnetic radiation resulting in molecular motion caused by migration of ions and rotation of dipoles^24^. It is of paramount importance to state that, higher temperatures ranging from 220 to 250 °C have been reported to cause deformation of the polytetrafluoroethylene (PTFE) vessels and have high risk of creating explosive conditions and therefore Teflon vessels were favoured^21^. The use of dilute H_2_O_2_ was preferred, because this reagent makes the proposed MA-HPD method to be greener, since high temperatures (above 150.2 ℃) of the microwave are known to convert H_2_O_2_ to hydrogen, oxygen and water^18^. The addition of 1 mL HNO_3_ was crucial, because the presence of H^+^ ions improve the extraction of metal ions, thereby increases the extraction recoveries^13^. In the current study, a known amount (0.1 g) of oil sample (crude oil, diesel, kerosene and gasoline), 9 mL of dilute H_2_O_2_ (5 M) and 1 mL concentrated HNO_3_ were transferred into 50 mL polytetrafluoroethylene-Teflon vessels (PTFE-TFM). Then, the samples were subjected to high temperatures (245 °C) of the microwave to break the metal carbon bond for easy extraction of the metal’s presence in the oil matrix. The digestion was allowed for 25 min, and the resulted digest were transferred to a 25 mL volumetric flask and filled up to the mark with Milli-Q water. Each of the samples was in triplicates with a blank as the fourth. The samples from the 25 mL volumetric flask ware then filtered using 0.45 µm pore diameter sized PVDF and transferred to a 15 mL centrifuge tube for analysis using the ICP-OES. The percentage recoveries (%R) of each element (Ba, Na, Ni and V) were also calculated using the Eq. 2 (Eq. 2). The experimental value was the concentration value obtained from the ICP-OES, divided by mass of sample, and multiplied by the dilution factor. The NIST 1634c value was the certified concentration written on the certificate of the certified reference material (SRM 1634c).

2 $$\%R=\frac{Experimental ~value}{NIST1634c~ value} \times 100\%$$

### Multivariate optimization

The multivariate optimization approaches were used for the determination of parameters that greatly affected microwave assisted hydrogen peroxide digestion (MA-HPD). The parameters that were optimized were digestion time, digestion temperature, sample mass and hydrogen peroxide concentration, these parameters were optimized using the full factorial design (2^n^). The variable was given the lower level (−) and the higher level (+), and the central point was not included in these experiments as presented in Table 2. The central composite design was used for further optimisation of the most significant parameters. For both two-level full factorial and central composite design, the Minitab 2018 statistical software was used for the generation of the experiments and analysis of data.

**Table 2:** The parameters investigated and their levels for two-level full factorial design.

Variable (factor optimised)	Low level (–)	High level (+)
Sample mass (g)	0.05	0.2
H_2_O_2_ concentration (M)	1	5
Digestion time (minutes)	20	60
Digestion Temperature (°C)	180	240

#### Full factorial design

A two-level full factorial design was used for the screening of optimised factors (sample mass, digestion time, digestion temperature and hydrogen peroxide concentration). The full factorial design was carried in way that the parameters were varied simultaneously for the optimised parameters. The 16 designed experiments generated by Minitab 2018 statistical software had the following ranges, temperature (180–240 °C), digestion time (20–60 min), sample mass (0.05–0.2 g) and H_2_O_2_ concentration (1–5 M) for the optimised parameters. It is worth noting to state that digestion was done to decompose the matrix making it to have minimal carbon content as high carbon cause plasma extinction during ICP-OES analysis^25,26^. The samples were taken for ICP-OES analysis after digestion and the results from ICP-OES were used to calculate the percentage recoveries for each element as per Eq. (2). Additionally, these recoveries were then run in Minitab 2018 statistical software to analyse the response of each parameter. The response of each parameter was expressed in form of Pareto chart and these parameters predicted the most significant factors.

### Microwave assisted acid digestion (MA-AD) a standard method

The mineralisation of the Reference Standard NIST1634c was digested under the same Anton Paar Multivariate 5000 microwave conditions. In a 50 mL PTFE-TFM vessel, approximately 0.1 g NIST1634c was weighed and 10 mL concentrated HNO_3_ was added. The vessels were then tightly sealed, and samples were subjected to high microwave temperatures for digestion. The temperature was ramped from room temperature to 245 °C in 10 min and it was held at the temperature for 15 min. It must be noted that the temperature was not 245 °C exactly in all vessels but it was in the range 245 ± 5 °C. Blanks were also digested in the same way as that of NIST1634c, however, in this condition the NIST1634c was not added (only concentrated acid was added). After digestion, the blanks and SRM samples were allowed to cool to room temperature, transferred to 25 mL volumetric flask and diluted to the mark. Filtering of digest was also done using 0.45 µm pore size PVDF before being transferred to 15 mL centrifuge tubes for ICP-OES analysis.

## Results and discussion

### Two level full factorial design

**Figure 1:**
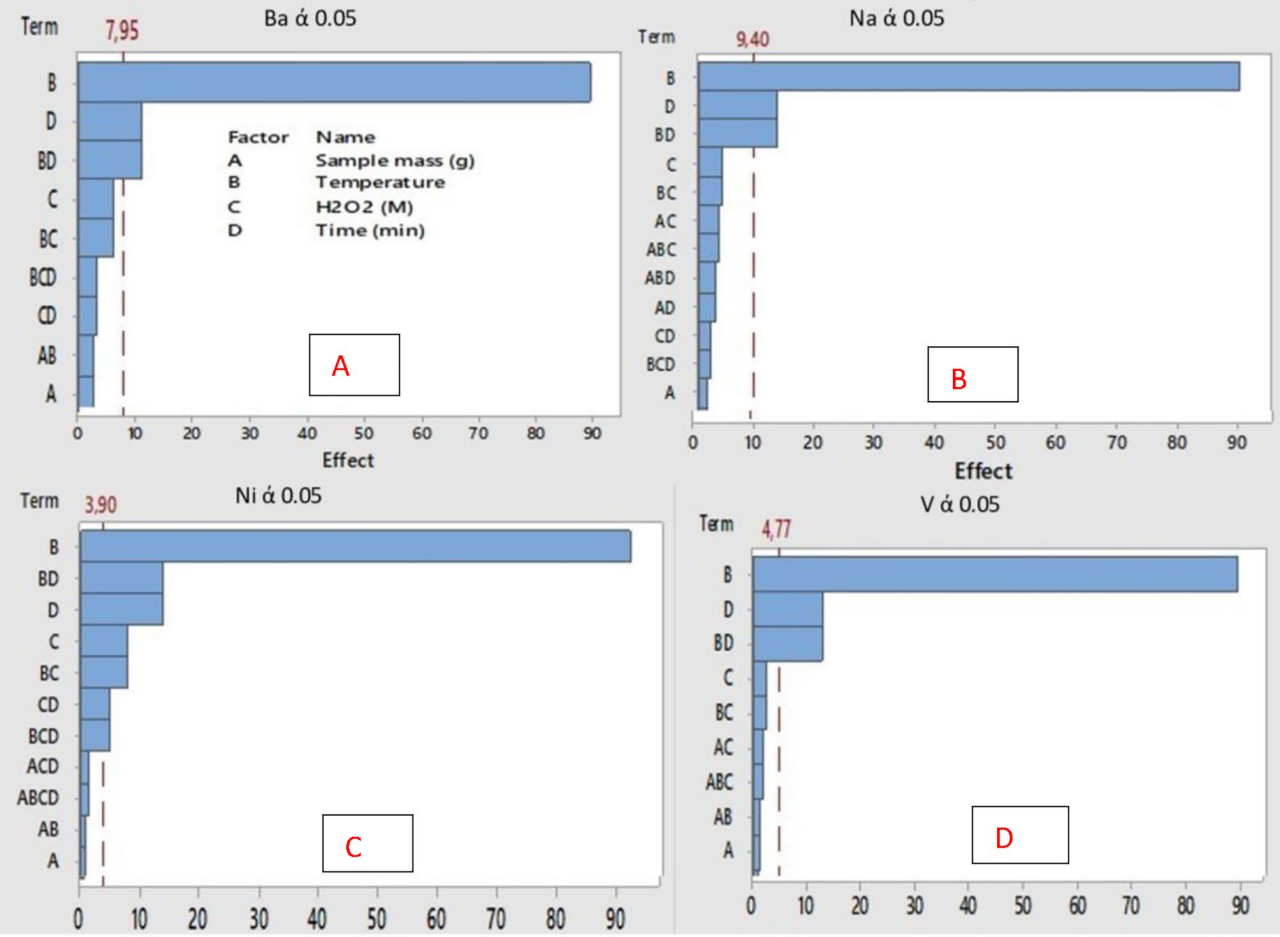
**(A-D)** Pareto chart for level 2 full factorial design (2^n^) at 95% confidence level for Ba, Na, Ni and V.

The screening process of the most influential factors of MA-HPD was achieved by using the two-level full factorial design (2^n^). The factors that were statistically significant were further optimised using the central composite design (CCD). The experimental results from the two-level full factorial design were examined by using the analysis of variance (ANOVA) at 95% confidence level (*p* = 0.05) and are presented in the form of Pareto charts for each metal as illustrated in Fig. 1A–D. The obtained results show that digestion time and digestion temperature ware statistically significant at 95% confidence level for the extraction of Ba, Na and V in NIST1634c digest. However, with Ni, in addition to digestion temperature and time, the concentration of H_2_O_2_ was observed to also be significant at 95% confidence level. For all the investigated metals, increasing digestion temperature and digestion time resulted in an increase in percentage recoveries. These observations agreed with Pareto charts, as they also showed digestion temperature as the most significant factor. The most significant parameters (digestion time and temperature) at 95% confidence level were then taken for further optimisation using the central composite design. It is worth noting that even though H_2_O_2_ concentration was significant for Ni recoveries, but it was not taken for further optimisation as it showed to be significant in only one metal ion.

### Response surface methodology (RSM)

Response surface methodology (RSM) are chemometric tools that helps in establishing quadratic models. These models assist to determine the critical conditions of factors under the study. Several RSM have been reported in literature which include BBD^27^, CCD^28^, three level factorial design^29^ and Doehlert matrix^30^. In the current study, CCD was selected for further optimization of digestion temperature and digestion time. The factors that were statistically insignificant, were kept at 0.1 g and 5 M for sample mass and H_2_O_2_ concentration, respectively. The 0.1 g sample was selected, because 0.05 g was giving poor precision (RSD ≥ 10%), while 0.2 g required longer digestion time (≥ 60 min). Alternatively, the choice of 5 M of H_2_O_2_ was based on the clear digests that were only observed when 5 M concentration level was used. The factors, number of experiments, experimental conditions, and results from the CCD are presented in Table S1.

**Figure 2:**
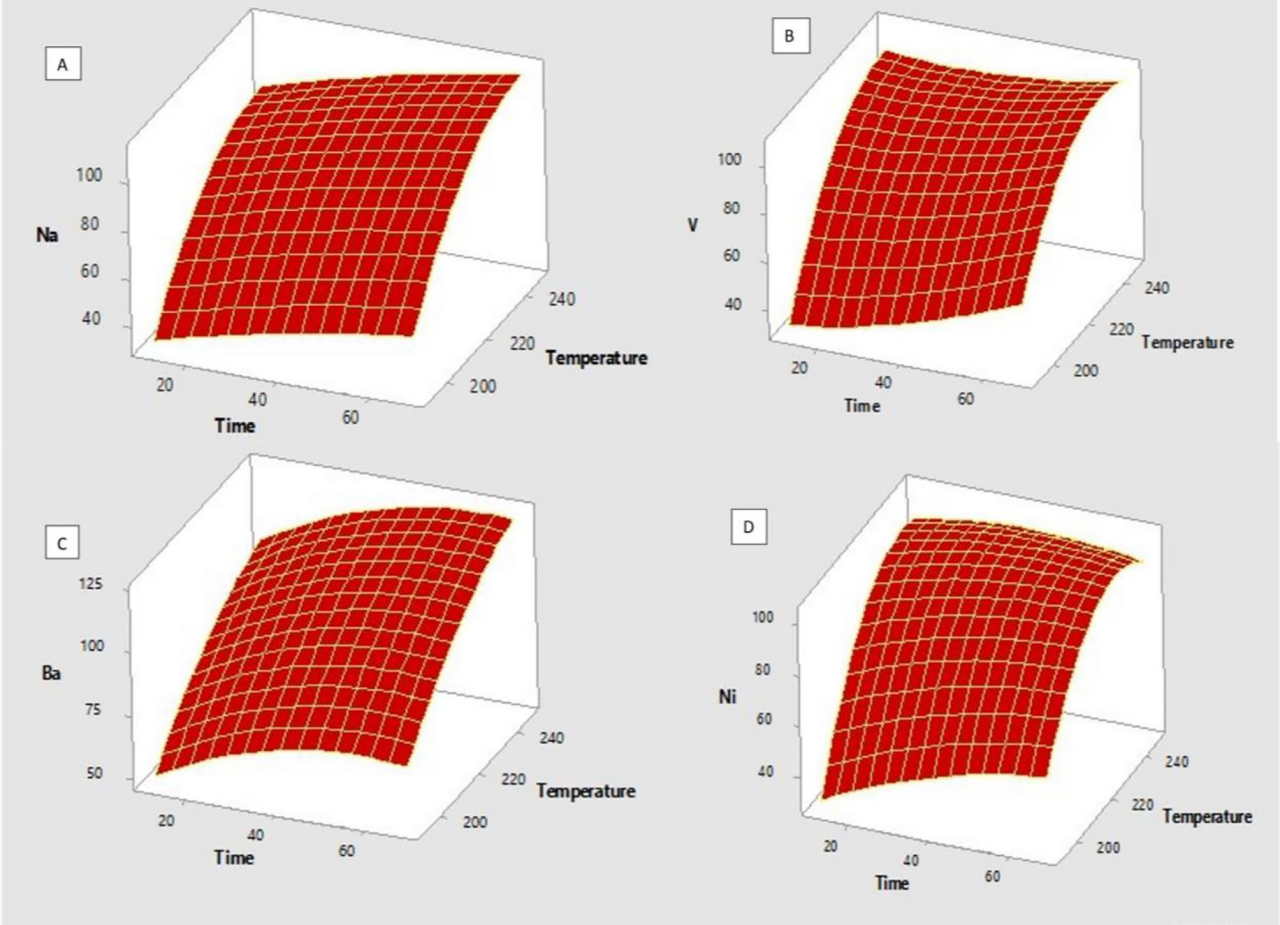
**(A-D)** Response surfaces for Na, V, Ba and Ni Versus Time. Temperature obtained from central composite design. Experimental conditions: 0.1 g of the sample and 5 M H_2_O_2_ While temperature and time were varied simultaneously (n = 3). Where n are number of replicates.

The response surface plots (Fig. 2A–D) were used to evaluate the effects of digestion time and digestion temperature on the extraction recoveries. The most optimum digestion conditions were predicted to be 0.1 g sample mass, 245 °C digestion time, 25 min digestion time and 5 M of H_2_O_2_ based on the quadratic equations and the surface plots obtained from the Minitab 18 software. The response optimiser was also used to predict the optimum conditions of microwave digestion and the findings were in agreement with quadratic equations and the surface plots results^31^. The response surface obtained from Minitab 2018 conditions indicated good accuracy of 106.8, 111.7, 90.0 and 109.8% for Ba, Na, Ni and V at 95% confidence level with precision of 2.9, 0.8, 2.7 and 4.1 for Ba, Na, Ni and V, respectively (results A). Then the same experiment was conducted, and the recoveries were 107, 117.7, 104 and 108.4% for Ba, Na, Ni and V, respectively while the standard deviation was found to be 1.6, 1.95, 4.66 and 3.33 for Ba, Na, Ni and V, respectively (results B). The small standard deviation between the seven experiments was a major proof of the reproducibility of the developed method (see Table S2). Therefore, results A and B were then compared using the t-test to verify if there were any statistical difference. The null hypothesis (Ho) means “there are no statistical difference between the two results’’, while the alternative hypothesis (H) means “there are statistical difference between the two results. The t-cal was 0.806 while t-tabulated was 1.943, this confirmed the acceptance of the null hypothesis, indicating that there was no statistical difference between results A and B at 95% confidence level. The same statistical analysis approach was reported by Manyangadze et al.^32^. The results for each analyte were further confirmed by the quadratic model (Eq. 3A–D), where A and B represent digestion time and digestion temperature, respectively. Additionally, the p < 0.05 was obtained in all the most significant factors which were time and temperature for all the metals. However, the lack of fit where the p > 0.05 was observed only in the two-way interaction of time*time and temperature *time. The analysis of variance (ANOVA) was also used to confirm if the data fits the model for Ba, Na, Ni and V at 95% confidence level. The p values for Ba, Na, Ni and V were 0. 076, 0.059, 0.073 and 0.052 respectively (See Table S3). This data indicated that the model perfectly fitted the data at 95% confidence level.

3A $$Ba=-561+0.92A+4.91B-0.1058{A}^{2}-0.00933B^2+0.0010AB$$

3B $$Na=-1047+0.39 A +9.17B-0.00265A2 -0.00933B2 + 0.00156AB$$

3C $$\mathrm{Ni}=-1530 + 2 506\mathrm{A}+13.37\mathrm{B}-0.0054\mathrm{A}2-0.02757{\text{B}}2-0.0831\mathrm{AB}$$

3D $$\mathrm{V}=-1327 + 1.44\mathrm{A}+11.56\mathrm{B}+ 0.00468{\text{A}}2-0.02339\mathrm{B}2-0.00694\mathrm{AB}$$

### Analytical figures of merit

For every developed analytical method, it is vital to determine analytical figures of merits which include method detection limits (MDL), method quantification limits (MQL) sensitivity, accuracy, precision, and correlation coefficient, just to name the few. These analytical merits help in drawing a conclusive decision on whether the newly developed method is better than the literature reported methods. In this study, the optimum conditions generated by the RSM were used for investigating the analytical features of the proposed MA-HPD method. This was achieved by digesting 0.025, 0.05, 0.1, 0.15, 0.2, 0.25 and 0.3 g of NIST1634c in triplicates and the digests were analysed using ICP-OES. The concentration of each metal in weighed mass (0.0, 0.025, 0.05, 0.1, 0.15, 0.2, 0.25 and 0.3 g) of NIST1634c CRM was calculated and plotted against intensity. The plotted graphs were able to provide information which included R^2^ and method calibration gradient (which is equivalent to the sensitivity of each metal). The metal that showed very high sensitivity was Na (2.03 × 10^5^ cps L mg^-1^) and the least sensitive metal was Ni (1.01 × 10^4^ cps L mg^-1^). The R^2^ ranged from 0,9992–0,9999 for all the metals (see Table 3). The standard deviation of 20 blanks was also obtained by digesting 20 blank samples (only 5 M of 9 mL H_2_O_2_ and 1 mL conc. HNO_3_). The gradient (slope) was then used to calculate limit of detection (LOD) and limit of quantification (LOQ) (see Eqs. 4 and 7). The LOD is referred to as the lowest concentration likely to be reliably distinguished from a blank sample and at which detection is feasible^33^. On the other hand, LOQ is referred to as the lowest amount of analyte in a sample which can be quantitatively determined with suitable precision and accuracy. The LOQ is equal to ten times the standard deviation of the blanks and all is divided by the method calibration (slope). The calculated LOD and LOQ were used to calculate the method detection limit and method quantification limit (see Table 3). Additionally, it is worth in noting that the LOD and LOQ provides the detection and quantification limits of the elements close to ideal conditions, where there are few other alloying elements. Since this happen in a very clean matrix, this LOD and LOQ are referred to as instrument detection and quantification limits, respectively. Therefore, the method detection limits and quantification limits were also calculated as these consider real-life matrices^34^.

**Table 3:** Analytical features of the MA-HPD method for quantitative extraction of Ba, Na, Ni and V in NIST1634c: Experimental conditions; microwave temperature (245 ^0^C), [H_2_O_2_] (5 M), sample amount (0.1 g), digestion time (25 min), replicates (n = 3).

Metal	SD of blank intensity (cps) (n = 20)	Sensitivity (cps L mg^-1^)	Accuracy (%)	Precision (%)	LOD (µg/L)	LOQ (µg/L)	MDL (µg/g)	MQL (µg/g)
Ba	4.6045	7.4 × 10^4^	107.1	2.9	0.187	0.622	0.046	0.155
Na	8.2014	2.03 × 10^5^	117.7	0.8	0.121	0.404	0.03025	0.101
Ni	5.513	1.01 × 10^4^	110.4	2.7	1.63	5.434	0.408	1.36
V	2.9983	3.92 × 10^4^	108.4	4.1	0.23	0.7658	0.057	0.1915

4 $$Limit ~of ~detection~ \left(LOD\right)=\frac{3*SD}{Method ~calibration ~slope}$$

5 $$Limit ~of~ quantification~ \left(LOQ\right)=\frac{10*SD}{Method ~calibration ~slope}$$

6 $$Method ~detection~ limit \left(MDL\right)=\frac{LOD*Final~ volume}{Optimum~ mass}$$

7 $$Method~ quantification ~limit~ \left(MDL\right)=\frac{LOD*Final ~volume}{Optimum ~mass}$$

### Comparison of the proposed MA-HPD with literature reports

The newly developed greener MA-HPD method was compared with other digestion methods in terms of its figures of merits and the findings are shown in Table 4. Shirlei al.^35^ reported a study on the digestion of crude oil for the determination of Ni and V prior to ICP-OES analysis. The reagents used for digestion were, 5 M of HNO_3_ and 4 M hydrogen peroxide. This method reported very low MDL of 237 and 60 ng/g for Ni and V, respectively. These results were almost in line with the ones reported in the current study (408 and 57 ng/g ) for Ni and V, respectively). However, when comparing the reagents used, the current study used a much environmentally friendly method as the 1 mL concentrated acid when put in the reagent solution was much dilute (1.58 M). Another study reported by Barela et al.^20^ on the digestion of biodiesel for determination of Ba, Co, Cr, Mn, Ni, Pb, Sr and V prior to analysis using SF-ICP-MS. This method reported very good accuracy (95–108%), precision (< 6%), and MDL (0.12, 2.8 and 0.12 ng/g) for Ba, Ni and V respectively. The newly developed method showed to be an alternative digestion method for mineralisation of fuel samples with reduced acidic secondary waste, because at high temperatures, H_2_O_2_ produces water and liberates oxygen which are environmentally friendly. In contrast, most literature reported mineralisation methods make use of high-volume concentrated acids, which resulted in the generation of carcinogenic nitrous oxide and secondary waste. The high concentration of produced nitrous oxide was reported to cause yellow permanent stains on digestion vessels, thereby reduces lifespan of the vessels.

**Table 4:** Comparison of MDL (µg/g), accuracy (%) and precision (% RSD) achieved by MA-HPD prior to analysis by ICP-OES with other literature reported digestion methods for Ba, Na, Ni and V in fuel samples: MAAD (Microwave assisted-acid digestion), MIC (Microwave induced combustion) and MA-HPD (Microwave assisted-hydrogen peroxide digestion ).

Fuel matrix	Sample preparation methods	Metal ions	Reagent	Concentration in real samples (µg/g)	Accuracy	Precision	MDL µg/g	Detection technique	Refs.
SRM 1634c	MA-HPD	Ba, Na, Ni and V	9 mL of 5 M H_2_O_2_ and 1 mL of conc. HNO_3_	0.55–58.86	104.8–117.7	≤ 4.1	0.046, 0.0304,0.408 and 0.057	ICP-OES	This work
Biodiesel	MAAD	Ba, Ni and V	10 mL of 7 M HNO_3_	0.0027–0.101	95–108	< 6	0.00012,0.0028 and 0.00012	SF-ICP-MS	^20^
Crude oil	MAAD	Mg, Pb and Sr	6 mL of 14.4 M of HNO_3_	Mg (12.5–201), Pb (0.09–0.203) and Sr (2.3–6.06)	95–104	< 4.0	0.1,0.3 and 0.07	MI-ICP-MS	^36^
Crude oil	MAAD	Na, Ca and Mg	4 M of H_2_O_2_ and 5 mL conc. HNO_3_	0.003–22.6	94–110	< 5	0.001–0.002	ICP-OES	^5^
Crude oil	MAAD	Ni and V	5 mL of 4 M H_2_O_2_ and 10 mL of 5 M HNO_3_	2–230	94.6–98.2	< 4.62	0.237 and 0.060	ICP-OES	^37^
Diesel	MAAD	Al, Cu, Fe, Ni and Ni	4 mL conc. HNO_3_ 5 mL H_2_SO_4_ and 5 mL H_2_O_2_	2- 25	70–78	< 5	0.052	ICP-MS	^11^
Fuel oil	MAAD	V	1 mL conc. HNO_3_and 1 mL H_2_O_2_	7.75–19.3	NA	< 5	0.250	GFAAS	^17^
Crude oil	MIC	Ni and V	6 mL of 5% H_2_O_2_ or 6 mL HNO_3_	(15.76–33.21) Ni and (1.07- 19.42) V	99–101	< 5	0.200 and 0.100	ICP-OES	^35^

**Figure 3:**
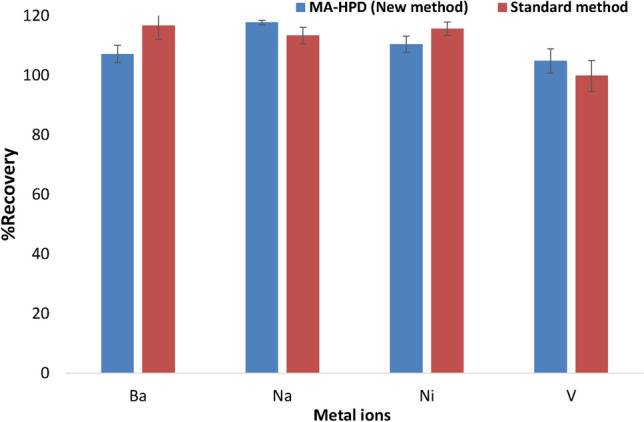
Comparison of percentage recoveries of target analytes (Ba, Na, Ni and V) when digestion was performed using dilute newly developed MA-HPD and standard classical MAAD methods.

The proposed environmentally friendly MA-HPD method was also validated by using Anton Paar Multiwave 5000 Microwave standard method for fuel samples (NIST1634c) and the ICP-OES results were compared (see Fig. 3). It is worth in noting that for the standard method, 10 mL of concentrated HNO_3_ was used while with MA-HPD, used 9 mL of 5 M H_2_O_2_ with 1 mL concentrated HNO_3_. The nitric acid was only used to enhance the extraction of metal ions from the decomposed organic matrix. It was observed that in terms of metal extraction recoveries (Ba, Na, Ni and V) there were no significance differences between the two digestion methods. However, the newly proposed method was advantageous as it is environmentally friendly (dominated by dilute H_2_O_2_).

The student *t*-test (at 95% confidence level, with ά = 0.05) was used for testing for any statistical differences between MA-HPD and MAAD based on analyte percentage recoveries of the two methods. The tabulated t was 3.182 indicating that for one to accept the null hypothesis calculated *t*-value must be within -3.182 < X > 3.182. The calculated *t*- value of 0.78974 was obtained. Therefore, the null hypothesis was accepted (H_0_ is the null hypothesis and H is the alternative hypothesis).

$$\begin{aligned} & H_{0} = {\text{No difference in the methods}}{.} \\ & H \ne {\text{There is a difference in the methods}}{.} \\ \end{aligned}$$

This therefore means that there were no statistical differences between the newly developed MA-HPD and the standard MA-AD methods which used concentrated acids, and for that reason the MA-HPD can be considered as an alternative method for digestion of fuel oils in the future.

### Application of MA-HPD in fuel sample

The optimised and validated MA-HPD method was then applied in real fuel samples (crude oil, diesel, gasoline, and kerosene). There were three samples for each fuel oil and were assigned as A, B and C. For the crude oil fractions (diesel, gasoline and kerosene), A, B and C meant different filling stations and the different labelling in crude oil samples meant different crude oil type. The samples were digested under the optimum conditions (250 ℃, 25 min, 9 mL of 5 M H_2_O_2_, 1 mL conc. HNO_3_ and 0.1 g sample) and analysed for the determination of Al, Ba, Cd, Co, Cr, Cu, Mg, Na, Ni, Pb, Sb, Ti, and V concentration levels using the ICP-OES. The concentration levels of the investigated metals are reported in Table 5. The latter has indicated that there is no significant difference in concentrations between the crude oil samples. However, it was observed that for most metals (Cr, Cu, Mg, Na, Ni and V) there was a decrease in concentration levels from the crude-oil to crude oil fractions. The reduction in concentration levels might have happened during crude oil refinery. However, Ba, Al and Pb showed increase in concentrations from the crude-oil to crude oil fractions. This might be due to corrosion of refinery and storage equipment of the diesel, gasoline and kerosene^24^. However, kerosene samples showed an increase in the levels of Al ranging from 26.8 to 47.0 µg/g and this increase might be caused by contamination from the Al based storage tanks. Moreover, Al residues in crude oils operate as catalyst poison during refining process, therefore its levels must be monitored, thereby controlled^38^.

**Table 5:** Concentration levels of metal ions expressed as µg/g in the real crude oil samples, diesel, gasoline and kerosene (A, B and C) after digestion using MA-HPD and analysis by ICP-OES.

Metal	Crude oil samples (µg/g)			Diesel samples (µg/g)			Kerosene samples (µg/g)			Gasoline samples (µg/g)		
	A	B	C	A	B	C	A	B	C	A	B	C
Al	2.36 ± 0.4	1.21 ± 0.2	2.13 ± 0.1	4.1 ± 0.04	36.75 ± 1.2	< DL	47.0 ± 1.2	< DL	26.8 ± 0.7	7.5 ± 0.05	6.5 ± 0.01	< DL
Ba	3.15 ± 0.2	1.83 ± 0.05	6.09 ± 0.05	7.7 ± 0.1	7.2 ± 0.9	9.2 ± 0.01	8.4 ± 0.1	6.2 ± 0.02	7.3 ± 0.1	8.3 ± 0.1	3.0 ± 0.03	9.2 ± 0.03
Cd	6.2 ± 0.1	6.8 ± 0.8	5.5 ± 0.07	4.9 ± 0.08	4.8 ± 0,03	4.99 ± 0.2	5.0 ± 0.05	4.9 ± 0.2	5.7 ± 0.09	5.1 ± 0.08	4.9 ± 0.02	5.0 ± 0.02
Co	12.6 ± 0.6	12.3 ± 0.2	14.07 ± 0.1	7.8 ± 0.09	8.8 ± 0.04	14.6 ± 0.5	14.6 ± 0.02	9.5 ± 0.2	16.7 ± 0.09	8.4 ± 0.3	9.4 ± 0.01	14.6 ± 0.01
Cr	7.39 ± 0.8	8.19 ± 0.08	8.49 ± 0.4	3.4 ± 0.02	2.9 ± 0.07	1.7 ± 0.03	1.8 ± 0.06	2.0 ± 0.06	1.1 ± 0.09	2.0 ± 0.001	1.5 ± 0.06	1.3 ± 0.006
Cu	2.05 ± 0.08	2.75 ± 0.04	2.89 ± 0.09	0.8 ± 0.002	0.8 ± 0.001	0.55 ± 0.001	0.56 ± 0.001	0.62 ± 0.001	0.6 ± 0.003	0.8 ± 0.002	0.86 ± 0.001	0.6 ± 0.001
Mg	47.4 ± 1.4	36.08 ± 1.1	47.39 ± 1.2	11.2 ± 0.1	10.3 ± 0.08	< DL	< DL	10.9 ± 0.2	< DL	11.2 ± 0.2	10.3 ± 0.8	< DL
Na	58.86 ± 1.1	52.7 ± 0.9	51.94 ± 1.6	18.7 ± 0.1	10.3 ± 0.1	34.3 ± 1.0	4.9 ± 0.07	2.5 ± 0.08	4.6 ± 0.07	34.3 ± 0.9	12.4 ± 0.1	35.1 ± 0.8
Ni	8.19 ± 0.08	6.14 ± 0.1	8.09 ± 0.1	2.4 ± 0.03	2.4 ± 0.02	4.9 ± 0,03	8.0 ± 0.02	2.2 ± 0.01	6.3 ± 0.06	4.9 ± 0.06	2.5 ± 0.08	4.6 ± 0.01
Pb	5.81 ± 0.1	5.06 ± 0.09	4.89 ± 0.06	2.7 ± 0.04	2.1 ± 0.01	8.0 ± 0.06	9.5 ± 0.1	1.6 ± 0.007	12.6 ± 0.6	2.0 ± 0.06	8.0 ± 0.01	2.0 ± 0.001
Sb	8.70 ± 0.2	3.37 ± 0.04	4.67 ± 0.03	1.9 ± 0.01	1.5 ± 0.07	9.5 ± 0.2	1.4 ± 0.08	1.5 ± 0.003	1.4 ± 0.001	1.4 ± 0.09	1.9 ± 0.02	1.6 ± 0.03
Ti	3.99 ± 0.05	4.42 ± 0.07	4.17 ± 0.08	1.5 ± 0.01	1.5 ± 0.05	1.5 ± 0.08	2.3 ± 0.1	2.5 ± 0.08	2.9 ± 0.08	1.4 ± 0.02	1.4 ± 0.08	1.4 ± 0.08
V	4.70 ± 0.06	4.67 ± 0.01	4.73 ± 0.04	1.9 ± 0.03	1.9 ± 0.03	2.0 ± 0.1	2.0 ± 0.05	1.9 ± 0.002	2.0 ± 0.06	2.1 ± 0.01	1.9 ± 0.06	2.0 ± 0.005

Despite the absence of Mg in station C of the crude oil fractions, it must be noted that the metals that reported high concentrations in overall samples included Mg (36.08–47.40 mg/kg) and Na (51.94–58.86 mg/kg). The presence of Na and Mg is not favoured, mostly in diesel and gasoline as these metals promote rapid deposit build up in vehicle engine which in turn can lead to deterioration of exhaust gas after treatment systems^36^. The concentration levels of Cu were very small in all the investigated filling stations. This is good, because Cu is mostly known for causing sediments and deposit formation resulting to clogging of automotive filters^39^. When considering the petroleum product acts of South Africa, a report was made on levels of Pb, Mn, K and P which was expected not to be above 13, 36, 10 and 14 mg/L in gasoline samples, respectively^40^. When looking at the levels of Pb in the fuel oils conducted in this study, it can be concluded that they are within the acceptable levels according to South African standards. When looking onto the in the premium unleaded (95 RON) that has been adopted by European countries which state that the levels of Pb must below 5 ppm, it can be concluded that the imported crude oil have acceptable levels for Pb. However, some of the crude oil fractions have Pb concentrations that were above 5 ppm, which might be due to contamination during transportation and storage of the diesel, kerosene, and gasoline. It is also worth mentioning that there is very little that has been reported on the legislation for fuel oils with regards to other metals and therefore most comparison was made based on the concentrations of metals determined in different studies.

The overall concentration levels of metals in crude oil, diesel, gasoline, and kerosene purchased in different felling stations of South Africa proved to be low in comparison to literature reports. For example, Shirlei et al*.*^35^ reported a study where determination of Ni and V was conducted in crude oil from three sampling sites of Brazil. These sampling sites reported Ni concentrations of 33.21, 15.76 and 29.52 mg/kg, which were much higher that the Ni concentrations (2.2–8.19 mg/kg) of the current study. For V, the concentrations were 1.07, 9.0 and 19.42 mg/kg which were also higher than the ones obtained from the current research (1.9–4.73). Mello et al.^41^ also reported the determination of Ni, V and S in Brazilian crude oil samples. The concentration of Ni and V ranged from 30.43 to 181 mg/kg and 36.9–763 mg/kg, respectively. Sant’Ana et al*.* reported a study on the mineralization of diesel samples prior to ICP-OES and the concentrations of the metals that were under study were 0.7–1 mg/kg (Al), 0.1–0.11 mg/kg (Cu), 0.36–0.41 mg/kg (Fe), 0.36–0.57 mg/kg (Zn) and Ni was found to be below the detection limit^11^. These concentrations were much lower in comparison to those reported in the current study for diesel. It is worth in noting that very little was reported on microwave -assisted digestion on crude oil fractions. Therefore, there was an urgent need to develop a mineralization method for determination of metal ions in South African fuel oils.

## Conclusion

The proposed microwave assisted-hydrogen peroxide digestion (MA-HPD) method showed very good accuracy: 104.8–117.7%, precision: ≤ 4.1% and MDL: 0.03 − 0.408 µg/g, for all the investigated metals. Even though 1 mL of HNO_3_ was added during mineralisation of the oils, this method was still cost-effective and green, because 9 mL dilute H_2_O_2_ converts to water at high temperature of the microwave and approximately 2.8% of the acid was left in the final digest of 25 mL. It is worthy to also indicate that metal ion standards for the calibration of spectrometric techniques are always preserved in an acidic environment of ≥ 1%. The multivariate optimisation tool was successful in determining the most optimum digestion conditions. The concentration levels of metals differed from one fuel matrix to the other. The majority of metals (Al, Ba, Cd, Cr, Cu, Ni, Pb, Sb, Ti and V) reported metal concentrations that were less than 10 µg/g. Additionally, for kerosene, an increase in levels of Al to a range of 26.8–47.0 µg/g was reported and this might be due to contamination in the two storage areas and the other fuel station for kerosene reported Al that was below detection limit. The levels of Cu were very low for all the fuel matrices, where in diesel, gasoline and kerosene were below 1 µg/g while in crude oil they were below 3 µg/g. Therefore, it can be concluded that the concentration levels of the investigated metals in South African fuel oils are not a threat.

### Supplementary Information

Supplementary Tables.

## Data Availability

All data generated or analysed during this study are included in this published article and its supplementary information file.
