# Supplementary material for: Microwave-assisted hydrogen peroxide digestion followed by ICP-OES for determination of metals in selected fuel oils
Source: Sci Rep. 2024 Jan 29;14:2362. doi: 10.1038/s41598-024-52898-4 (PMC10824727; doi:10.1038/s41598-024-52898-4)
Supplement: Supplementary file 1 — Supplementary Tables. [file 41598_2024_52898_MOESM1_ESM.docx]

**Microwave-assisted hydrogen peroxide digestion followed by ICP-OES for determination of metals in selected fuel oils**

Njabulo S. Mdluli^1^, Cyril D. Knottenbelt^2^ Philiswa N. Nomngongo^3^, Nomvano Mketo^1*^

*^1^Department of Chemistry, College of Science and Engineering and Technology, Florida Science Campus, University of South Africa, Roodepoort, 1710, Johannesburg, South Africa*

*^2^PetroSA, Private bag X14, Mossel Bay, 6520, South Africa*

*^3^Department of Chemical Sciences, University of Johannesburg, PO Box 17011, Doornfontein 2028, Johannesburg, South Africa*

**Table S1**: The effect of varying digestion time and temperature while keeping sample mass and H_2_O_2_ concentration constant in further optimization using on the digestion of central composite design. Experimental conditions: 0.1g of the sample and 5M H_2_O_2_ (n =3).

| **Temp °C** | **Time (Min)** | **Percentage Recovery** | | | | **% RSD** | | | | |
| --- | --- | --- | --- | --- | --- | --- | --- | --- | --- | --- |
|  |  | **Ba** | **Na** | **Ni** | **V** | **Ba** | **Na** | **Ni** | **V** |  |
| 220 | 40 | 115.7 | 88.6 | 89.5 | 83.3 | 1.8 | 0.3 | 2.4 | 5.2 |  |
| 220 | 40 | 109 | 87.0 | 92.5 | 88.7 | 2.4 | 0.6 | 2.6 | 4.7 |  |
| 220 | 40 | 108.8 | 81.3 | 92.9 | 85.2 | 1.6 | 0.6 | 2.3 | 5.3 |  |
| 240 | 60 | 104.0 | 100.0 | 104.4 | 99.2 | 2.2 | 1.0 | 1.8 | 4.1 |  |
| 191 | 40 | 73.0 | 52.5 | 49.0 | 39.3 | 4.9 | 5.1 | 4.9 | 7.1 |  |
| 200 | 20 | 78.8 | 56.7 | 68.4 | 69.3 | 5.1 | 6.9 | 7.1 | 8.9 |  |
| 200 | 60 | 76.0 | 69 | 90 | 82.6 | 4.4 | 4.4 | 4.2 | 6.4 |  |
| 248.3 | 40 | 110 | 109 | 102 | 109.8 | 2.9 | 0.8 | 1.9 | 4.1 |  |
| 220 | 40 | 108.8 | 81.3 | 92.9 | 85.5 | 09 | 3.1 | 2.0 | 4.8 |  |
| 240 | 40 | 111.3 | 87.7 | 92.6 | 98.3 | 2.1 | 2.7 | 1.6 | 3.9 |  |
| 220 | 40 | 102 | 82 | 87 | 83 | 1.8 | 2.1 | 1.7 | 4.7 |  |
| 220 | 11.7 | 80 | 74 | 68 | 61 | 6.1 | 0.9 | 1.9 | 4.9 |  |
| 220 | 68.3 | 106.2 | 106.2 | 108.1 | 103.3 | 4.1 | 0.7 | 2.4 | 5.4 |  |

**Table S2**. Percentage recoveries obtained from the seven-method validation experiments

| **Experiment** | **Studied metal and their percentage recoveries (%) per experiment** | | | | |
| --- | --- | --- | --- | --- | --- |
|  | **Ba** | | **Na** | **Ni** | **V** |
| Exp 1 | | 108 | 117.0 | 106 | 109 |
| Exp 2 | | 106 | 114.8 | 108 | 112 |
| Exp 3 | | 108 | 115.0 | 98 | 104 |
| Exp 4 | | 108 | 119.8 | 96 | 110 |
| Exp 5 | | 106 | 118.8 | 1081 | 104 |
| Exp 6 | | 104 | 119.2 | 108 | 113 |
| Exp 7 | | 109 | 119.3 | 104 | 107 |
| mean | | 107 | 117.7 | 104 | 108.4 |
| SDV | | 1.60 | 1.95 | 4.66 | 3.33 |

**Table S3 (A-D).** Analysis of variance for the response surface methodology for Ba, Na, Ni and V

(A) Analysis of Variance for Ba

| **Source** | **DF** | **Adj SS** | **Adj MS** | **F-Value** | **P-Value** |
| --- | --- | --- | --- | --- | --- |
| Model | 5 | 2706,33 | 541,27 | 22,29 | 0,000 |
| Linear | 2 | 1554,89 | 777,44 | 32,02 | 0,000 |
| Temperature | 1 | 1352,67 | 1352,67 | 55,71 | 0,000 |
| Time | 1 | 202,22 | 202,22 | 8,33 | 0,023 |
| Square | 2 | 1151,01 | 575,51 | 23,70 | 0,001 |
| Temperature*Temperature | 1 | 717,21 | 717,21 | 29,54 | 0,001 |
| Time*Time | 1 | 582,89 | 582,89 | 24,01 | 0,002 |
| 2-Way Interaction | 1 | 0,42 | 0,42 | 0,02 | 0,899 |
| Temperature*Time | 1 | 0,42 | 0,42 | 0,02 | 0,899 |
| Error | 7 | 169,96 | 24,28 |  |  |
| Lack-of-Fit | 3 | 134,50 | 44,83 | 5,06 | 0,076 |
| Pure Error | 4 | 35,47 | 8,87 |  |  |
| Total | 12 | 2876,29 |  |  |  |

(B) Analysis of Variance for Na

| **Source** | **DF** | **Adj SS** | **Adj MS** | **F-Value** | **P-Value** |
| --- | --- | --- | --- | --- | --- |
| Model | 5 | 3158,67 | 631,73 | 8,71 | 0,006 |
| Linear | 2 | 2944,13 | 1472,07 | 20,29 | 0,001 |
| Temperature | 1 | 2715,96 | 2715,96 | 37,44 | 0,000 |
| Time | 1 | 228,17 | 228,17 | 3,15 | 0,119 |
| Square | 2 | 123,34 | 61,67 | 0,85 | 0,467 |
| Temperature*Temperature | 1 | 98,28 | 98,28 | 1,35 | 0,283 |
| Time*Time | 1 | 13,46 | 13,46 | 0,19 | 0,680 |
| 2-Way Interaction | 1 | 91,20 | 91,20 | 1,26 | 0,299 |
| Temperature*Time | 1 | 91,20 | 91,20 | 1,26 | 0,299 |
| Error | 7 | 507,75 | 72,54 |  |  |
| Lack-of-Fit | 3 | 456,28 | 152,09 | 11,82 | 0,059 |
| Pure Error | 4 | 51,47 | 12,87 |  |  |
| Total | 12 | 3666,42 |  |  |  |

(C) Analysis of Variance for Ni

| **Source** | **DF** | **Adj SS** | **Adj MS** | **F-Value** | **P-Value** |
| --- | --- | --- | --- | --- | --- |
| Model | 5 | 2515,15 | 503,03 | 17,06 | 0,001 |
| Linear | 2 | 1744,21 | 872,11 | 29,58 | 0,000 |
| Temperature | 1 | 1736,18 | 1736,18 | 58,88 | 0,000 |
| Time | 1 | 8,04 | 8,04 | 0,27 | 0,618 |
| Square | 2 | 559,23 | 279,62 | 9,48 | 0,010 |
| Temperature*Temperature | 1 | 394,18 | 394,18 | 13,37 | 0,008 |
| Time*Time | 1 | 102,98 | 102,98 | 3,49 | 0,104 |
| 2-Way Interaction | 1 | 211,70 | 211,70 | 7,18 | 0,032 |
| Temperature*Time | 1 | 211,70 | 211,70 | 7,18 | 0,032 |
| Error | 7 | 206,39 | 29,48 |  |  |
| Lack-of-Fit | 3 | 197,94 | 65,98 | 31,24 | 0,073 |
| Pure Error | 4 | 8,45 | 2,11 |  |  |
| Total | 12 | 2721,54 |  |  |  |

(D) Analysis of Variance for V

| **Source** | **DF** | **Adj SS** | **Adj MS** | **F-Value** | **P-Value** |
| --- | --- | --- | --- | --- | --- |
| Model | 5 | 3364,46 | 672,89 | 10,78 | 0,003 |
| Linear | 2 | 2609,11 | 1304,55 | 20,90 | 0,001 |
| Temperature | 1 | 2606,49 | 2606,49 | 41,75 | 0,000 |
| Time | 1 | 2,61 | 2,61 | 0,04 | 0,844 |
| Square | 2 | 698,35 | 349,18 | 5,59 | 0,035 |
| Temperature*Temperature | 1 | 215,53 | 215,53 | 3,45 | 0,106 |
| Time*Time | 1 | 394,83 | 394,83 | 6,32 | 0,040 |
| 2-Way Interaction | 1 | 57,00 | 57,00 | 0,91 | 0,371 |
| Temperature*Time | 1 | 57,00 | 57,00 | 0,91 | 0,371 |
| Error | 7 | 437,03 | 62,43 |  |  |
| Lack-of-Fit | 3 | 421,16 | 140,39 | 35,39 | 0,052 |
| Pure Error | 4 | 15,87 | 3,97 |  |  |
| Total | 12 | 3801,49 |  |  |  |
